# Beyond national indicators: adapting the Demographic and Health Surveys’ sampling strategies and questions to better inform subnational malaria intervention policy

**DOI:** 10.1186/s12936-021-03646-w

**Published:** 2021-03-01

**Authors:** Ifeoma D. Ozodiegwu, Monique Ambrose, Katherine E. Battle, Caitlin Bever, Ousmane Diallo, Beatriz Galatas, Manuela Runge, Jaline Gerardin

**Affiliations:** 1 Department of Preventive Medicine and Institute for Global Health, Northwestern University, Chicago, IL USA; 2 Institute for Disease Modeling, Seattle, WA USA; 3 Global Malaria Programme, World Health Organization, Geneva, Switzerland

## Abstract

In malaria-endemic countries, prioritizing intervention deployment to areas that need the most attention is crucial to ensure continued progress. Global and national policy makers increasingly rely on epidemiological data and mathematical modelling to help optimize health decisions at the sub-national level. The Demographic and Health Surveys (DHS) Program is a critical data source for understanding subnational malaria prevalence and intervention coverage, which are used for parameterizing country-specific models of malaria transmission. However, data to estimate indicators at finer resolutions are limited, and surveys questions have a narrow scope. Examples from the Nigeria DHS are used to highlight gaps in the current survey design. Proposals are then made for additional questions and expansions to the DHS and Malaria Indicator Survey sampling strategy that would advance the data analyses and modelled estimates that inform national policy recommendations. Collaboration between the DHS Program, national malaria control programmes, the malaria modelling community, and funders is needed to address the highlighted data challenges.

## Background

The growing spatial and temporal variability in malaria risk [1–3], increasing diversity in malaria control tools[4], and limited funding availability is precipitating the need for malaria-endemic countries to adopt intervention policies that move away from a one-size-fits-all approach to one that is specifically tailored to their subnational context. The Global Technical Strategy for malaria (GTS) recommends that targeted strategies be country-led [5]. The High Burden to High Impact (HBHI) initiative further describes how each country can drive its strategy with its own data, including routine health facility reporting, national household surveys, and post-campaign assessments that collect information on current gaps in intervention coverage [6]. Mathematical modelling can be used to integrate these data sources together to predict the impact of possible subnational intervention strategies and explore whether achieving a strategic malaria target is feasible.

Many National Malaria Control Programmes (NMCPs) are now choosing to target interventions at the district level (second administrative level, admin-2), given that this is an operationally feasible unit at which intra-provincial heterogeneity is captured. Malaria endemic countries are typically federations comprised of varying numbers of admin-2 units or districts located within a first administrative level (admin-1, also called states or provinces). While the seat of subnational government is usually at the admin-1 level, admin-2 units are officially delineated geographical areas with some form of self-government to decentralize the running of local affairs. Monthly reports from health facilities provide routine surveillance data that can be aggregated up to admin-2 units. Routine data is used to identify malaria trends and needs at the local level [7, 8], as recommended by the GTS.

Challenges with data quality and completeness as well as lack of sufficient historical data and delayed reporting hinder the use of routine data to form a rigorous understanding of the country’s malaria past and present situation and intervention needs. Because these datasets only include individuals who seek treatment at reporting health facilities, they provide no insight into individuals who live in less-accessible areas or who seek treatment from private and informal health care sectors. The incomplete view of malaria incidence and treatment provided by routine reporting can result in biased estimates of population burden and access to care.

National surveys, such as the Demographic and Health Surveys (DHS) and the Malaria Indicator Surveys (MIS), supplement routine surveillance by providing representative estimates of malaria prevalence and intervention coverage. Initiated in 1984, the DHS was originally designed to collect comparable population-based data on indicators of sexual and reproductive health, maternal and child health, and nutrition in low and middle-income countries (LMICs) [9]. As funding for malaria programmes increased, it became necessary to continue to make the investment case for additional funding with local data, giving rise to the inclusion of a malaria module in the DHS in 1999 and the introduction of the MIS in 2006 [10]. National health strategic planning in LMICs only considered aggregated indicator estimates at the admin-1 level; as such, the sampling methodology of DHS and MIS were devised to capture health status, services and interventions at that level. The shift towards accounting for district-level health and intervention indicators within subnational malaria strategies calls for refreshing the DHS and MIS sampling strategy and questionnaires to meet the needs of national programmes.

In their current form, both the DHS and MIS have many strengths that support health decision making. The DHS and MIS capture a wide range of health indicators allowing comprehensive assessment of a country’s health situation. As part of their multistage design, survey participants are selected from clusters and households within a fully covered geographic sampling frame, offering researchers the opportunity to examine how ecological and individual-level factors relate to the distribution of health outcomes. Moreover, survey questionnaires are standardized to enhance the comparability of indicators across populations and time. In malaria specific programs and research, analyses by NMCPs and the research community generate insight into spatial and temporal differences in malaria indicators, which allow data-driven prioritization of intervention deployment and serve as parameters for mathematical models. This makes the DHS and MIS an important resource for NMCPs and the global health community.

Given the limitations of routine surveillance, NMCPs and modellers use the DHS to understand the subnational malaria context. NMCPs increasingly consider outputs of mathematical models when planning sub-national malaria strategy, including making decisions about expansion of chemoprevention and choosing from a set of vector control strategies. To address related questions, epidemiological models must capture historical trends in transmission, current patterns of exposure, and intervention coverage for each subnational area. This piece highlights how DHS and MIS data are utilized by mathematical models and suggest improvements that would enhance both modelling and data analysis efforts from NMCPs to facilitate informed decision-making. For the sake of brevity, the term “DHS” is used to encompass both DHS and MIS surveys.

## DHS data is useful for national policy-making but parameterizing subnational malaria transmission models is challenging

Models of malaria transmission used for national strategic planning are informed by household survey data on intervention coverage, transmission intensity, and malaria burden. To set subnational intervention coverages, models rely on DHS measures of treatment-seeking rates for febrile illness among children under five, insecticide-treated nets (ITN) usage at the household level and for different age groups, and coverage of intermittent preventive treatment in pregnancy (IPTp). Modelled transmission intensity can then be calibrated to capture DHS measures of the *Plasmodium falciparum* parasite rate in children under the age of five (*Pf*PR_0–5_).

**Fig. 1:**
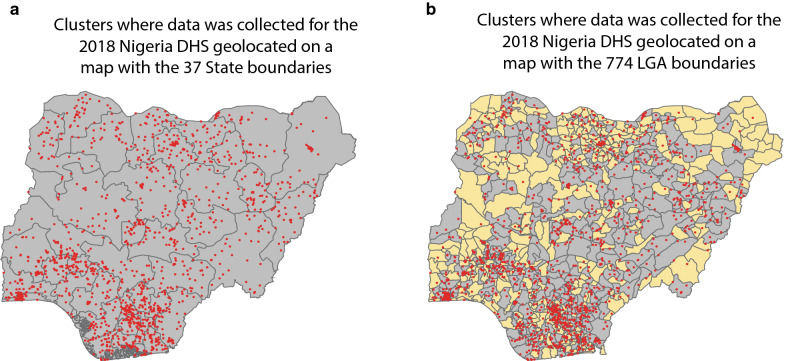
**a** State-level (admin-1) map of Nigeria with red dots representing clusters where DHS data collection was conducted in 2018. The number of clusters in state boundaries range from 20 to 54 with a median of 36. **b** Local government area (LGA)-level (admin-2) map of Nigeria with red dots representing clusters where DHS data collection was conducted in 2018. LGAs colored in yellow are areas where estimation of malaria indicators will be challenging because they contain zero or one cluster. Number of clusters within LGA boundaries ranged from zero to 11 with a median of two. 103 LGAs had no clusters

**Fig. 2:**
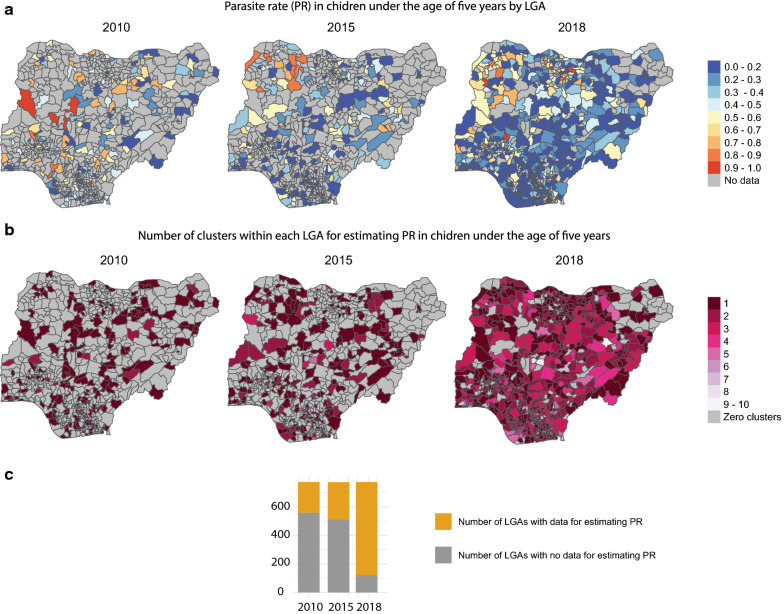
**a** *Pf*PR_0-5_ according to 2010, 2015, and 2018 Nigeria DHS. The LGA prevalence values depicted are not representative for the population per LGA, as DHS is not powered at the LGA level. **b** Number of clusters located within each LGA boundary used to estimate *Pf*PR_0–5_ in each DHS year. Many LGAs contain zero DHS clusters, although geographic coverage improved substantially in the most recent DHS. **c** Number of LGAs with and without PR data. LGAs without data were 558 in 2010, 510 in 2015 and 121 in 2018, out of 774 total LGAs

Using the DHS to parameterize fine scale models introduces additional sources of uncertainty. To help NMCPs stratify and plan operations, models must capture data at admin-2. However, estimates of malaria prevalence and intervention coverage from the DHS are only meant to be representative at a state or provincial (admin-1) level (Figs. 1, 2) and are underpowered to measure these indicators at the admin-2 level. Modelling predictions based on parameters from DHS household cluster data would, therefore, be biased. Moreover, data collection and cleaning standards for georeferenced DHS data also increase the risk of biased admin-2 projections. Sampling errors while using GPS receivers to georeference cluster locations could lead to attribution of admin-2 data from one to another. Additionally, the displacement of cluster locations to protect participants’ confidentiality [11] and any resultant random effects from data jittering would further exacerbate the problem of misclassifying admin-2 data.

Malaria indicators captured by the DHS are subject to seasonal variations in malaria transmission and human behaviour, which limit understanding of malaria transmission intensity, ITN use, and comparability of yearly surveys. Parasite rate is typically at its maximum during the rainy or peak mosquito-biting season and trends downwards in the dry season. Individuals use ITNs during the wetter months and reduce usage in the dryer months when mosquito activity is diminished [12]. Treatment-seeking behaviour can be affected by seasonal accessibility issues and seasonal demands on parents’ time, for example, agricultural needs during the wet season. Therefore, malaria indicators from DHS surveys conducted during the dry season months do not necessarily capture parasite rate, ITN use, and case management coverage in the peak transmission season. Surveys conducted in different seasons, even within the same DHS year, are not directly comparable without adjustment for the seasonality effect. NMCPs and modellers resort to other data sources with a narrower geographic scale to capture seasonal and temporal changes in malaria transmission and accurately identify gaps in intervention coverage and areas of high prevalence.

The restriction of current questions to select age groups limit how informative the results are for driving country strategy and parameterizing models. For example, the DHS only tests children under the age of five for malaria infection, which, although important, is of limited utility for categorizing malaria transmission intensity in settings where more of the burden is in older children or adults. *Pf*PR_0-5_ measured during implementation of seasonal malaria chemoprevention (SMC) may be particularly uninformative as *Pf*PR is suppressed in this population and SMC coverage is not assessed in the DHS. Measurements of *Pf*PR in older children can be more informative than *Pf*PR_0–5_ even in high-transmission areas, as children above age two will have some immunity to clinical malaria, and hence less treatment with anti-malarials, yet limited immunity to parasitaemia itself [13]. Some models, therefore, apply standardization algorithms to convert *Pf*PR_0–5_ to *Pf*PR_2–10_ [14]_._ While such algorithms have been validated in prior work [13], the extent of bias introduced by predicted *Pf*PR_2–10_, especially in fine-scale models, is unknown.

A similar issue arises with using the DHS data to evaluate case management and treatment coverage for uncomplicated malaria, where questions are restricted to children under the age of five. NMCPs, therefore, know little about access to malaria treatment in older children, where burden is increasingly shifting [15]. In the absence of case management information for uncomplicated malaria in older children and adults, modellers either assume homogeneous coverage by age or turn to site-specific research studies on treatment-seeking behaviour.

**Fig. 3:**
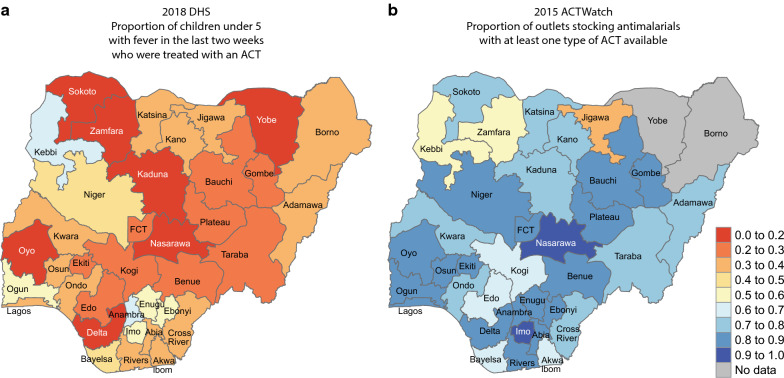
**a** Nigeria’s 2018 DHS shows the proportion of children with fever in the 2 weeks preceding the survey that were treated by with an ACT. **b** Proportion of outlets stocking anti-malarials that had at least one type of ACT based on the 2015 ACTWatch survey [16, 17]. Maps share the same legend

Estimating case management rates from DHS data requires analysing questions directed at a subset of DHS participants, which reduces the sample size and may introduce validity issues and inconsistencies. In the 2018 Nigeria DHS, effective case management coverage, that is the proportion of children under the age of five that received artemisinin-based combination therapy (ACT) among those that had a fever within the 2 weeks prior to the survey, was 22% at the national level. Disaggregated at the state level, ACT-related case management was remarkably low in many areas. For example, the 2018 DHS suggests that febrile children were not treated at all with ACT in Nasarawa, and only about 3 to 4% in Zamfara and Yobe (Fig. 3a).

When these estimates were discussed with the Nigerian National Malaria Elimination Programme, they indicated that the actual ACT use would likely be higher than that seen in the 2018 DHS, and the state level DHS estimates would not agree with their perceived ACT use in many parts of the country. The 2015 ACTWatch survey [16, 17] supports this view, which indicated that most outlets stocking any anti-malarials in individual states had at least one type of ACT medicine for sale (Fig. 3b). While the metrics are clearly different, the ACTWatch data suggests intense penetration of ACT across both the public and private health care sectors in Nigeria, and, together with the Nigerian programme perspective, calls into question the 2018 DHS results that suggested extremely low rates of artemisinin-based combination therapy in some areas of Nigeria. This discrepancy of trends between access (ACTWatch) and use (DHS) metrics emphasizes the limitations of the current DHS sampling strategy to capture case management coverage among febrile children, who are few in number, and the need for a strengthened DHS data collection system that builds trust and meets NMCP needs.

The gaps that we have identified within the DHS sampling strategy and questionnaires do not diminish the immense contribution of the DHS Program to evidence-based decision-making. However, when DHS measures do not adequately capture malaria indicators, or DHS data are out of concordance with institutional knowledge and beliefs of intervention and treatment access and malaria risk behaviour, deciding where to target interventions becomes more challenging and a data-driven approach nearly impossible.

## Recommendations

Having outlined the major barriers to using the DHS for evidence-based subnational malaria strategic planning, we propose changes to the DHS surveys and sampling strategy to improve understanding of the malaria context at the relevant spatial scale of programmatic decision-making and drive more accurate predictions of the impact of targeted interventions.

1. **Time DHS surveys to capture malaria indicators during the high-transmission season**. Carefully timing the DHS survey to coincide with the high malaria transmission season and collecting data at the same time every year would improve estimation of malaria indicators, and comparability of yearly surveys, even at finer scales. Effective timing provides understanding of transmission intensity and intervention coverage at its peak periods and implies that a smaller sample size would be needed to accurately estimate malaria indicators. Hence, if the DHS retains a similar sampling strategy but conducts surveys only in the wetter months, malaria indicators will be more precisely estimated at both admin-1 and admin-2-level, and NMCPs can more reliably track indicator trends. Suppose accessibility issues preclude peak-season surveys in some districts, a hybrid approach where isolated districts are surveyed at a different time of year may be necessary.
2. **Support malaria-endemic countries to conduct admin-2-level and/or monthly surveys**. We hope to see the DHS support malaria-endemic countries in conducting more frequent and granular surveys at the admin-2 level. Increasing the spatial resolution of the survey to the admin-2 level will enhance the precision of survey estimates, and if these admin-2 surveys are done monthly, it would lead to excellent understanding of seasonal and temporal changes in parasite rate and intervention coverage. At the outset, priority could be given to districts in high-transmission areas where intervention targeting will be most beneficial, or surveys could be conducted only during high-transmission months. The survey’s frequency and scale could be reduced if low spatial and temporal heterogeneity in malaria indicators are detected within neighboring districts. While we acknowledge that this comes with higher survey implementation costs for the DHS, this will vary for individual countries depending on several factors including coverage of the existing DHS survey, the number of admin-2 areas, and the target population sizes. The extra implementation costs will be relatively lower for some countries and possibly unfeasibly high in others. However, the potential savings from allocating resources to the most-at-risk population and thereby additional lives saved could serve as a justification for increased funding for the DHS to pursue a broader sampling strategy.
3. **Extend blood smear or rapid diagnostic testing (RDTs) to children up to the age of 10 years**. As prevalence in the youngest children declines, testing of older children will be more informative for assessing malaria transmission intensity. In lower-transmission areas, collection of prevalence in adults will become necessary to identify remaining areas of sustained transmission for intervention targeting. Deprioritizing blood smears in favour of RDTs can help mitigate some of the added expense.
4. **Adapt the DHS sampling strategy and survey questions to better capture data that inform estimation of treatment coverage**. To obtain an improved estimate of treatment modalities, we recommend the DHS oversample children in selected high transmission settings where case management with ACT is particularly crucial to prevent death. The current DHS sampling approach may not provide accurate estimates of artemisinin-based combination therapy rates for malarial fevers, which lessens its utility in intervention planning. If recommendation #1 is adopted, the additional sample would not be substantial since the sampling frame for febrile children will be significantly enhanced. Qualitative research is needed to better understand how to word questions around care-seeking and access to effective treatment, as this could be a limiting factor in the accuracy of participant responses, and understanding where the cascade of care falls apart is necessary for identifying solutions to low treatment rates. Questions on case management urgently need to be extended to older children and adults so that policy makers understand how symptoms and treatment dynamics vary by age, time, and transmission intensity in their country.
5. **Add questions to the DHS to capture data on SMC coverage**. In many areas with highly seasonal malaria transmission, SMC is a crucial intervention to reduce malaria incidence and mortality during the high-transmission season. DHS surveys implemented during peak transmission months would be well-positioned to measure SMC coverage, which is often challenging for NMCPs to calculate from doses distributed due to uncertain population denominators. Measuring SMC coverage will enable NMCPs to better assess implementation quality and to identify gaps and will allow models to generate more accurate predictions of the impact of SMC expansion or changes in scheduling.
6. **Leverage the Service Provision Assessment (SPA) surveys to monitor malaria incidence and case management, even if at an aggregate level**. The SPA surveys, which are part of the DHS portfolio of surveys, provide country-specific overviews of health service delivery. SPA surveys can be leveraged to obtain a snapshot of reported malaria incidence, severity, and case management modalities at the time of the DHS community surveys. This information can be very powerful: NMCPs can contextualize effective treatment results from the survey, and modellers can triangulate data from both surveys to capture and explain transmission dynamics.
7. **Make the DHS dynamic and flexible to adapt to a changing intervention landscape**. The landscape of malaria interventions is heterogeneous and can change with new strategic plans and pilots of intervention deployments. DHS design should be cognizant of local interventions. In areas where new interventions are introduced, survey questions related to the interventions can be asked only in those administrative units. Likewise, if interventions are discontinued in a particular locality, survey questions can be modified in response.

## Conclusion

The DHS is already an invaluable tool for informing malaria intervention strategies and could be an even greater asset for subnational planning if the changes we propose are made to augment the existing DHS platform. We call for a dialogue between the DHS Program experts, NMCPs, the malaria modelling community and funders to discuss existing data challenges and design a practical path for overcoming them. As countries move toward geographically tailored national strategies, the need for high-quality information is paramount. The experience and technical expertise of the DHS Program is essential to meet this need.

## Data Availability

All data used in this work are publicly available. DHS is available for download from https://dhsprogram.com/. ACTwatch data is available for download from https://malariaatlas.org/actwatch/.
